# Design and basic research on accuracy of a novel individualized three-dimensional printed navigation template in atlantoaxial pedicle screw placement

**DOI:** 10.1371/journal.pone.0214460

**Published:** 2019-04-02

**Authors:** Xiao-Long Chen, Ya-Fen Xie, Jian-Xin Li, Wu Wu, Guan-Nan Li, Hui-Jing Hu, Xiao-Yun Wang, Zhao-Jian Meng, Yue-Feng Wen, Wen-Hua Huang

**Affiliations:** 1 Department of Spine Surgery, Guangdong Province Work Injury Rehabilitation Hospital, Guangzhou, China; 2 Department of Joint Surgery, Guangdong Province Work Injury Rehabilitation Hospital, Guangzhou, China; 3 Institute of Rehabilitation medicine, Guangdong Province Work Injury Rehabilitation Hospital, Guangzhou, China; 4 Department of Radiotherapy, Affiliated Cancer Hospital & Institute of Guangzhou Medical University, Guangzhou, China; 5 National Key Discipline of Human Anatomy, School of Basic Medical Sciences, Southern Medical University, Guangzhou, China; University Magna Graecia of Catanzaro, ITALY

## Abstract

**Objective:**

To design and evaluate the accuracy of a novel navigation template suitable for posterior cervical screw placement surgery by using 3D printing technology to improve the existing guiding template design.

**Methods:**

The researchers (including spine surgeons and technicians) used CT to perform thin-slice scanning on 12 cases of normal upper cervical vertebral specimens and defined the screw channels that were completely located in the pedicle without penetrating the cortex as ideal screw channels, then designed the ideal channel of the upper cervical vertebral (atlantoaxial) pedicle screw by computer software which was regarded as the preset values, and recorded the screw entrance point, transverse angle and sagittal angle of the ideal channel. Then, researchers designed the novel navigation templates for placement pedicle screw according to the ideal screw channel preset values and manufactured them with one for every single vertebra by 3D printer. A senior spine surgeon performed the posterior surgery to implant pedicle screw on the specimens by the novel navigation templates, then performed CT thin-slice scanning on the specimens again after removing the screws, and reconstructed the actual screws channel by computer software, recorded the screw entrance point, transverse angle and sagittal angle of the actual channels which were defined as the actual values and evaluated them according to Kawaguchi’s pedicle screw evaluation standard finally. The differences between the preoperative preset values of ideal screw channel and the postoperative actual values of actual screw channel were compared by a nonparametric paired rank test.

**Results:**

48 screws were placed on 12 cases of upper cervical vertebral specimens in total. It showed that the grade 0, I, II, III channels in this study were 47, 1, 0, 0, respectively. The grade 0 channels accounted for 97.92% of the total number of channels. There was no significant difference with regard to the screw entrance point, the transverse angle, and the sagittal angle between the preoperative preset values of ideal screw channels and the postoperative actual values of actual screw channels.

**Conclusion:**

To implant pedicle screw assisted with the novel individually navigation template designed by 3D printed in the posterior cervical surgery can improve accuracy of pedicle screw placement and safety of the surgery.

## Introduction

Posterior placement of pedicle screw fixation is an important treatment strategy for some upper cervical traumas and diseases, such as fractures, joint dislocation complicated with neurological symptoms. To place the pedicle screw accurately and successfully is the key to the success of the operation. However, the application for posterior placement of pedicle screw fixation is confined by the high risk of vascular and nerve damage due to the complex adjacent structure of the atlantoaxial spine.

In recent years, with the application of additive manufacturing (3D printing) technology in the medical field, the placement of pedicle screw assisted by 3D printing navigation templates have been gradually applied to spinal surgery because of its high accuracy [1–3]. However, most of the existing navigation templates have guiding channels, which require a larger surgical space during operation and make it more difficult to place the navigation templates, and to adhere the surface of guiding templates closely to the bone surface. On the other hand, the fixed directional guiding channels limit the flexibility to adjust the direction of the screw channel during the operation. All of the design drawbacks above result in its inconvenience in use, and limit its clinical application. Therefore, it is necessary to create a novel navigation template with the improvement on the design of the navigation template. The new navigation template removed the guiding channel of the original guiding template and retained only the screw hole. At the same time, the original navigation channel was replaced by an inward-moving navigation pole. This research evaluated the new navigation template accuracy in the auxiliary screws placement to ensure its effectiveness.

## Materials and methods

### Specimens/Cases

This study was in line with the spirit of the Helsinki Declaration and approved by the Ethics Committee of the Guangdong Provincial Work Injury Rehabilitation Hospital. The human corpse anatomic preservative specimens were obtained from the Institute of Clinical Anatomy of Southern Medical University.

The selected specimens should met the following inclusion criteria: (1) age ≥ 16 years old, (2) the neck was free from deformity and injury, and its bone structure was intact.; (3) imaging examination showed that Atala-Dens Interval (Atala-Dens Interval, ADI) ≤ 5mm, or the space Available for the Cord (SAC) ≤ 13mm; (4) The structure itself had not been damaged by surgery or trauma; and (5) X-ray densitometer was normal without osteoporosis.

The research enrolled 12 cases of normal atlas and axis anatomical preservation specimens that met the inclusion criteria. From June 2018 to August 2018, posterior screw placement in upper cervical was performed in the laboratory of the Institute of Rehabilitation Medicine of rehabilitation hospital. The specimens included 7 males and 5 females, with the age range from 28 to 63 years. All the instruments and pedicle screws were provided by Kangli Orthopedic Medical Devices Co., Ltd. (Jiangsu, China) and the screw placement was performed by the same surgeon.

### Design improvement and production of 3D printed navigation template

Imaging examinations were performed by using the SIEMENS 64-slice spiral CT scanner (SIEMENS Healthcare, Munich, Freistaat Bayern, Germany). At least three segments of the 1st-3rd cervical vertebrae were scanned by CT with the parameters as follows: 1.0 mm in thickness, 0.65 mm in pitch, 1 turn/second in tube speed, 130 kV in voltage, and 104 mA/s in current. The scanned images were saved in a medical format for digital imaging and communication (DICOM).

**Fig 1:**
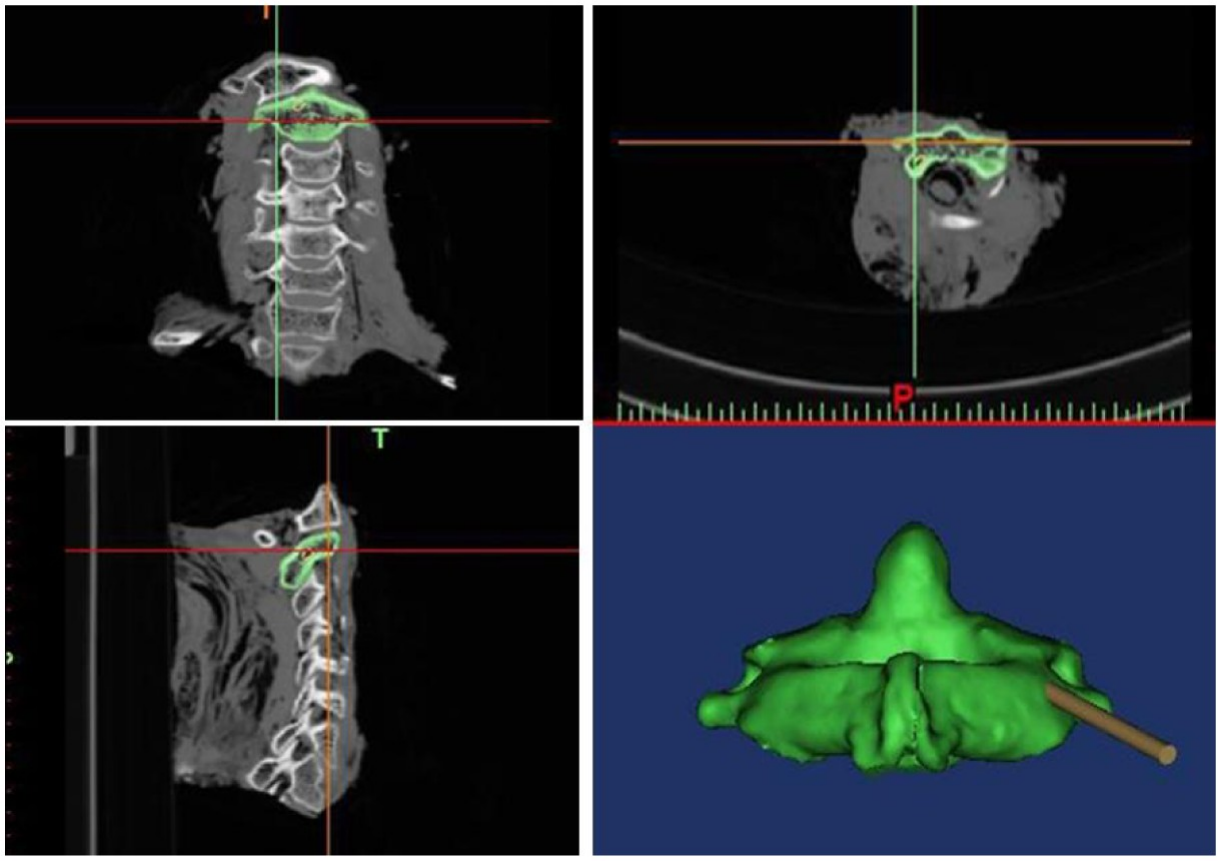
The 3D model of the target cervical spine (axis).

The DICOM images were imported into 3D image generation and editing processing software (Mimics 17.0, Materialise, Leuven, Belgium) to generate a 3D model of the target vertebra. In the sagittal plane, coronal plane, horizontal plane and 3D overall compositions, the cylindrical parts of 3 mm in diameter were imported (Fig 1). Through the dynamic monitoring of each section, the scientific research placed the cylindrical parts in the proper position. The screw channel (analysis body) did not penetrate cortical bone, was completely inside the pedicle without passing through the vertebral artery foramen and the spinal canal, which were defined as ideal pedicle screw channels for computer-designed perfect surgery (Fig 1). The “sagittal angle” was defined as the angle between the pedicle screw and the sagittal plane. The "transverse angle" was defined as the angle between the screw path and the horizontal plane. In this research, the determination of the screw entrance point was actually achieved by measuring the distance from the screw point to a fixed point of the atlantoaxial. The fixation point of atlas was defined as the apex of the posterior nodule of posterior arch. The fixed point of the axis was the apex of the root of spinous process. Then the preoperative design data such as "sagittal angle" in the sagittal plane, "transverse angle" in the horizontal plane and the screw entrance point was recorded.

**Fig 2:**
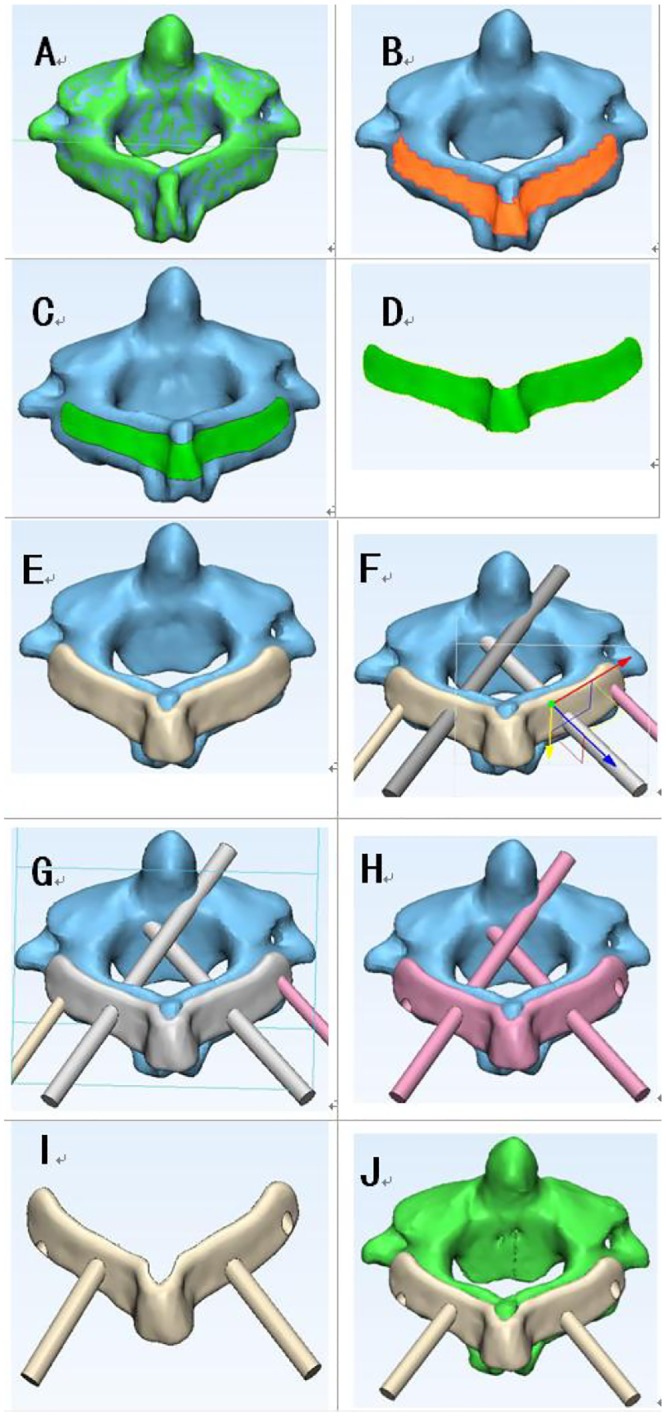
Images show the design process of novel navigation template. (A) “Wrap”. (B) "Zone Mark". (C) "Smooth". (D) Extracted shape. (E) "hollow". (F) Imported cylindrical parts. (G) Formed a guiding pole. (H) Formed screw hole. (I)The 3D model of novel template. (J). Matched the vertebral body.

**Fig 3:**
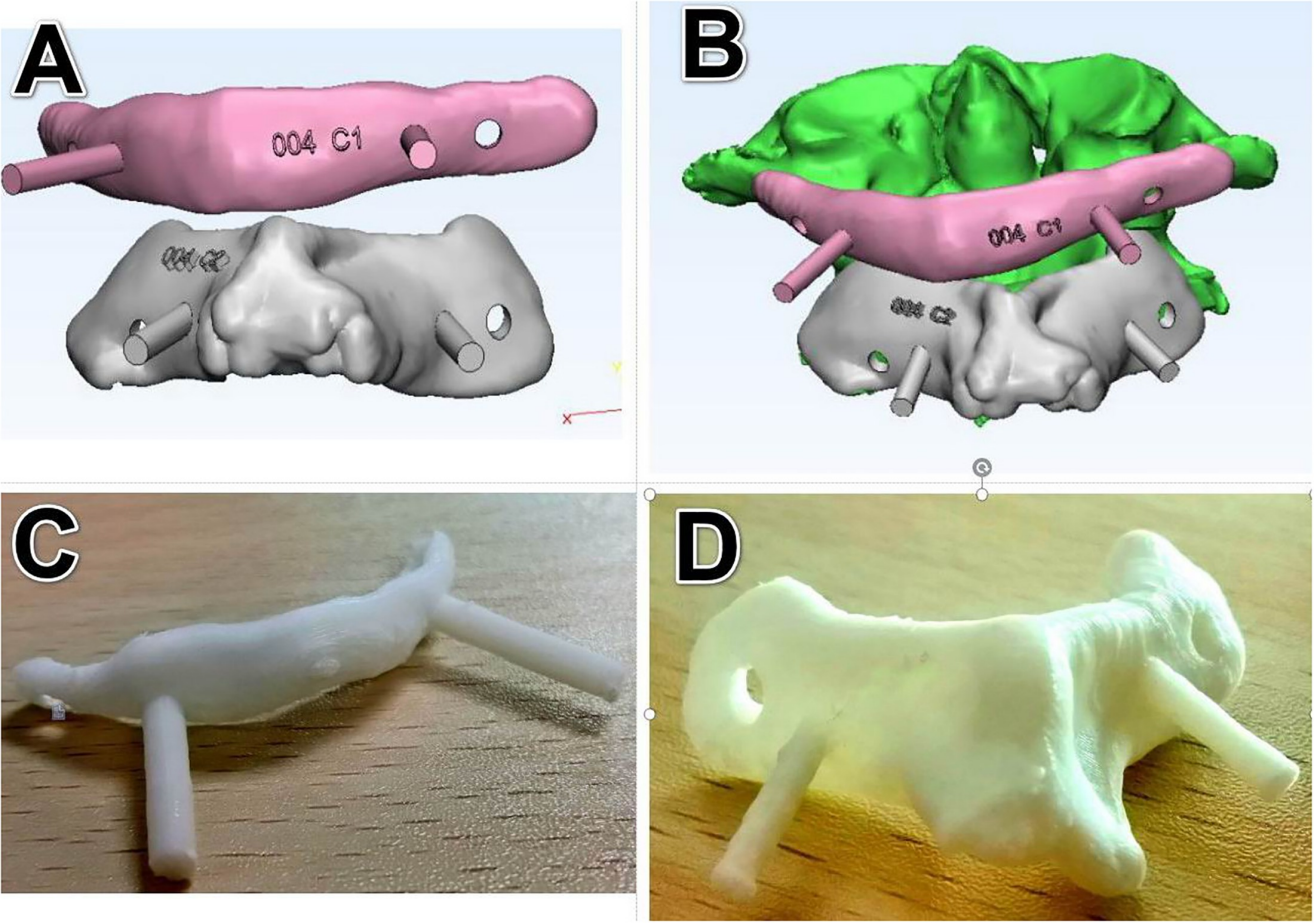
The atlantoaxial 3D model and printed product of navigation template. (A-B) The atlantoaxial 3D model. (C-D) The novel atlantoaxial navigation template.

After the screw channels were designed, the boffins performed the “wrap” operation on the generated three-dimensional body (3-Matic 9.0, Materialise, Leuven, Belgium) (Fig 2A), closed various micropores of the bone surface, and then performed "Zone Mark" operation on the posterior part of the three-dimensional body (Fig 2B), including the surface of lamina and the spinous process by Mimics equipped with the 3-matic design software. The edge of the marked area was "smoothed" (Fig 2C and 2D) and then the area was propagated by the "hollowing" (Fig 2E) function to form the contact part of navigation template. A new pair of 4mm diameter cylinders was copied from the materialised cylindrical parts (Fig 2F). In the range of the contact part of the template, the left and right new cylinders were shifted inward about 1.5 cm. Using the Boolean addition operation, the new cylinders were merged with the navigation template to form a guiding pole/ handrail(Fig 2G). Then the interface between the navigation template and the three-dimensional model was removed by Boolean subtraction operation to form the contact surface of the guiding template. At last, the original diameter 3mm cylinder was removed by Boolean subtraction to form a screw hole (Fig 2H). The design of the novel screw placement navgation template complete (Fig 2I and 2J). The experted .stl file of the navigation template 3-Matic design results were imported into the Formlabs 3D printer (Formlabs, Inc, Somerville Massachusetts, USA). The parameters of novel navigation template were set: the thickness of the guiding template was 1 mm, the diameter of the navigation pole was 3 mm, the length of the navigation pole could be 3–6 cm; the diameter of the screw hole was 4 mm, and the gap between the guiding template and the bone was 0. Then PLA (polylactic acid) material was used to print out a novel type of atlantoaxial pedicle screw guiding template, and some rough parts were polished to make it smooth and ready for subsequent usage (Fig 3A–3D).

### Surgical procedure

**Fig 4:**
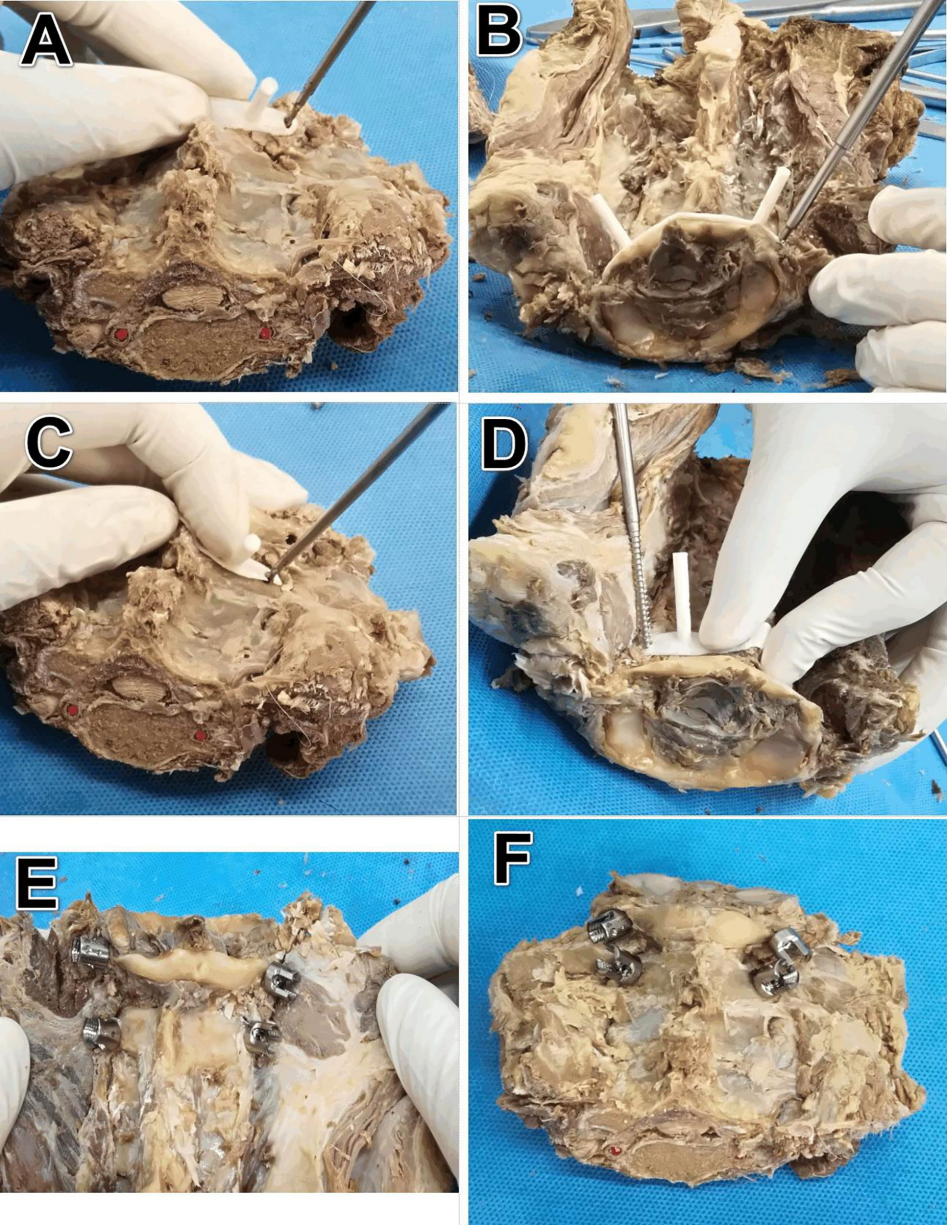
Pedicle screw placed by navigation template in atlantoaxial surgery. (A-B) Placed screw in the atlas. (C-D) Placed screw in axis. (E-F) The situation after completely implanted screw.

The researchers used 3D printed navigation template to assist the screws placement on human corpse specimens. The specimens were placed in the prone position to simulate the intraoperative situation. The scientific personnel performed the median incision on the posterior neck and separated the paraspinal muscles layer by layer to reveal the posterior bony structure of the atlantoaxial [4], then attached the navigation template tightly to the surface of the posterior bony structure of the corresponding vertebra after dissecting the soft tissue like muscles and ligaments of the posterior vertebra structure through holding on the guiding pole/ handrail of navigation template. At last, the navigation poles were fixed after confirming for they were tightly fit. The researchers used a hand-drill with 2.0 mm in diameter to open the channel from the screw hole and then drill into the bone from all directions monitoring under direct vision, so that the drill bit was kept parallel to the direction of the guiding pole. The C-arm X-ray machine fluoroscopy was used to determine the drilling depth routinely. After examing on the bone integrity of the inner wall of the screw channel without any defect by the probe and confirming the correct adhesion degree and no displacement of the navigation template, the pedicle screws with a diameter of 3.5mm were implanted into the screw channel which was tapped by the wire (Fig 4A–4F).

### Evaluation methods

**Fig 5:**
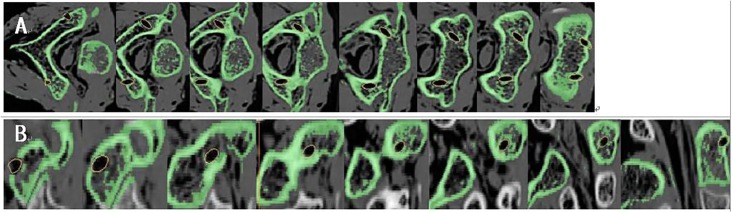
Monitor the pedicle screw channel position in sagittal and horizontal planes. (A) The screw channel position from horizontal plane. (B) The screw channel position from sagittal plane.

**Fig 6:**
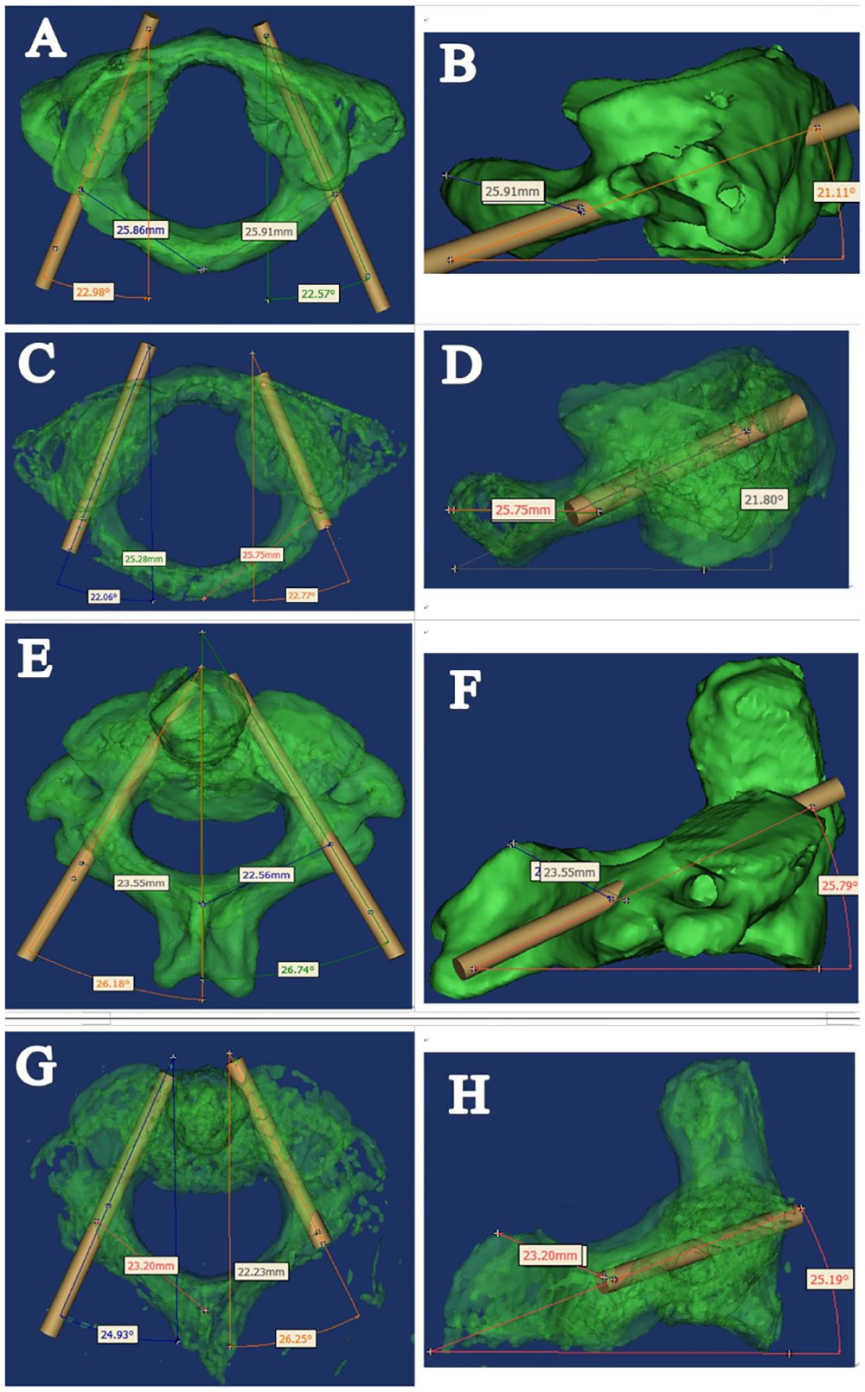
Reconstructed the screw channel and compared the differences between preoperative and postoperative data. (A-B) The preoperative screw channel in atlas. (C-D) The postoperative screw channel in atlas. (E-F) The preoperative screw channel in axis. (G-H) The postoperative screw channel in axis.

The accuracy of the atlantoaxial pedicle screw was evaluated by a author (C.L.) who did not participate in the surgery. After removing the screws of all of the specimens, CT scans were performed on them again with the parameters consisted with the last time scans. After importing the postoperative CT scan data into Mimics software, The boffins observed the pedicle screw channel position from the sagittal, coronal and horizontal planes (Fig 5A and 5B), and then re-introduced the cylindrical parts according to the position to show the postoperative actual screw channel of the vertebra (Fig 6C, 6D, 6G & 6H). The accuracy of screw placement was evaluated according to Kawaguchi et al [5]: grade 0, the screw was completely located in the vertebral pedicle; grade I, the screw penetrated the pedicle bone cortex <2 mm without complications; grade II, the screw penetrated the pedicle bone cortex >2 mm without complications; and grade III, complications related to screw placement occurred, such as nerve and vertebral artery injuries. Grade 0 was considered to be the correct location of pedicle screws and safe placement.

The formed three-dimensional images were processed by "transparency" to observe the actual channel after the screws placement. The scientific research observed the actual screw entrance point and the transverse angle on the horizontal plane from the top view, as well as the sagittal angle from the left and right sagittal planes respectively, and measured them by the Mimics software’s measurement function as postoperative data (Fig 6C, 6D, 6G & 6H).

### Statistical analysis

IBM SPSS Version 22.0 (IBM Corp., Armonk, New York, USA) was used for statistical analysis. Since the data in the research were non-normally distributed, so the nonparametric paired rank test was selected. The difference between the preoperative and postoperative data of sagittal and transverse angle, and the screw entrance point were compared with a nonparametric paired rank test. A two-sided P-value greater than 0.05 was considered statistically significant.

## Results

### Postoperative evaluation of general results

The screw placement on all of the specimens was successfully completed by using a novel individualized 3D printed navigation template. The average operative time and screw placement time was 37.67±6.83 minutes and 3.68±0.99 minutes/piece, respectively (Table 1). The CT image showed that except for one grade I screw channel, all of the screw channels were grade 0 screw channels in which screws did not penetrate the outer cortex.

**Table 1:** Perioperative parameters.

Perioperative Parameter	Navigation Template
average operative time (min)	37.67±6.83
average screw placement time (min)	3.68±0.998

### Evaluation of accuracy of screw placement

**Fig 7:**
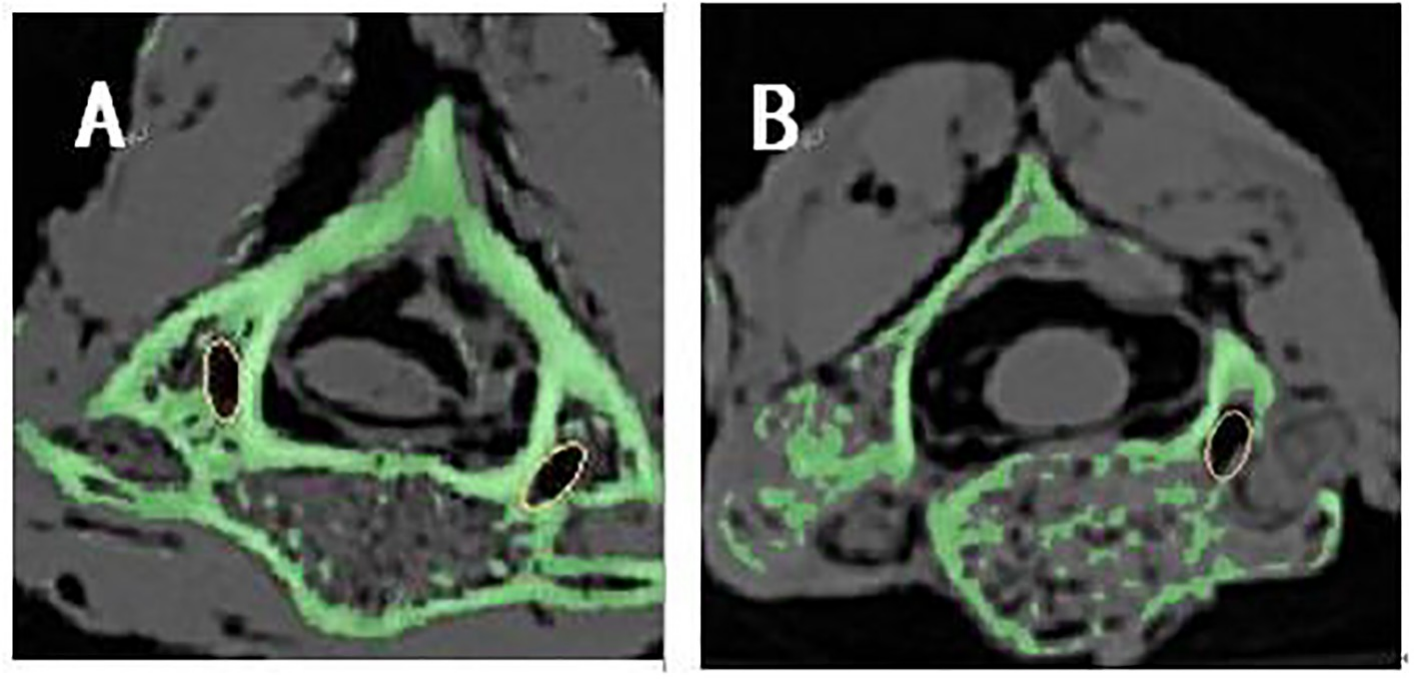
Accuracy evaluation of pedicle screw placement by Kawaguchi method. (A) Grade 0 screw channel. (B) Grade I screw channel.

All specimens were scanned by CT after the operation. 48 pedicle screws (24 in C1 and 24 in C2) were placed in 12 specimens by using the novel individualized 3D printed screws placement navigation templates. The screw placement accuracy evaluation according to Kawaguchi’s criterion[5] showed that in this study all the screw channels were grade 0 except for 1 case with grade I (Fig 7A and 7B), and none of the screw channels were gradeII or III (Table 2). The differences between the preoperative preset ideal value and the postoperative actual value of the sagittal and transverse angle, and the screw entrance point were measured and compared by the Mimics software. There were no significant differences in average transverse and sagittal angle, and the screw entrance point between the preoperative preset and the postoperative actual screw trajectories for atlas on the left and on the right (Table 3). Regarding the axis, no significant differences were also observed in average transverse and sagittal angle, and the screw entrance point for atlas on the left and on the right (Table 4).

**Table 2:** Accuracy atlantoaxial pedicle screw placement by Kawaguchi evaluation method.

	Preoperative Ideal group	Postoperative Actual group
Number of Grade 0	48	47
Number of Grade I	0	1
Number of Grade II	0	0
Number of Grade III	0	0
Total Number of Screws	48	48
Accuracy	100%	97.91%*

*Statistically significant difference compared with preoperative Ideal group (P > 0.05).

**Table 3:** Comparison of Tansverse and sagittal Angles, and screw entrance point between preoperative ideal and postoperative actual screw Trajectories in C1.

	Preoperative Preset Angle		Postoperative Actual Angle	
	Ideal (Left)	Ideal (Right)	Actual (Left)	Actual (Right)
Sagittal Angle (°)	21.12±5.13	22.70±3.98	21.39±4.82*	23.53±4.09*
Tansverse Angle (°)	25.65±5.65	24.44±2.40	25.33±5.51*	24.76±2.31*
Screw Entrance Point (mm)	27.41±3.79	25.76±2.03	27.29±3.74*	25.95±2.19*

*Statistically significant difference compared with preset angle (P > 0.05).

**Table 4:** Comparison of Tansverse and sagittal Angles, and screw entrance point between preoperative ideal and postoperative actual screw Trajectories in C2.

	Preoperative Preset Angle		Postoperative Actual Angle	
	Ideal (Left)	Ideal (Right)	Actual (Left)	Actual (Right)
Sagittal Angle	21.29±5.99	24.18±3.31	21.16±7.04*	23.53±3.71*
Tansverse Angle	27.46±8.17	27.26±9.56	27.32±7.98*	27.28±9.39*
Screw Entrance Point	24.69±3.03	24.99±2.07	24.63±3.04*	25.12±2.13*

*Statistically significant difference compared with preoperative preset angle (P > 0.05).

## Discussion

In many cases, posterior atlantoaxial screw fixation is the gold standard for the treatment of atlantoaxial fractures and dislocations. Due to the complex adjacent structures of the upper cervical spine, traditional surgery has a high risk of vascular and nerve injuries [6, 7]. It is reported that the incidence is more than 29–47% of which the screws break through the bony cortex during the upper cervical screw placement. In recent years, it was reported that a computer-assisted navigation screw placement method could improve the accuracy and safety of screw placement, reducing the risk of spinal nerve and blood vessel damage [8]. However, the high cost of navigation equipment, the preoperative "registration" step, and the intraoperative "drift" can lead to prolonged operation time and hinder the "perfect" implantation of pedicle screw. Due to these reasons, this method is not widely used in clinical practices.

In spinal surgeries, based on the individual data of atlantoaxial CT imaging, the ideal pedicle screw channel for each patient can be designed by using 3D printing technique, which was regard as "perfect" screw placement. In order to improve the accuracy of screw placement, an ideal pedicle screw placement scheme can be given by using the printed screw navigation templates. 3D printing navigation template has the following advantages: (a) individualized pedicle screw channel design has high accuracy of screw placement, (b) no prior experience is required to assist the operator in performing the "fool" operation, and (c) all the guiding plates are designed independently for every single vertebra, so it will not happen that other or entire pedicle screw channels have error positioning due to the guiding template introperative position change. By using 3D navigation templates, Lu et al [9] implanted 84 pedicle screws in human cervical vertebrae, of which 82 screws were placed in grade 0 (97.6%), and only 2 screws were grade I (2.4%). Kawaguchi et al [5] used 3D guiding templates to place cervical pedicle screws in 42 cases, of which 42 cases screw insertion were grade 0 (95.4%), and only 2 cases were grade I (4.6%). There were neither grade II or III cases, nor neurovascular injury related complications.

There are some influential factors in 3D printing screw navigation templates, such as the quality and thickness of the slice of the CT scan, and the process of extracting and modifying the vertebra model on the computer may affect the accuracy of the guiding template or lead to defaults in making it. During the operation the screw channel deviation is prone to occur, if the soft tissue like muscles and ligaments of the posterior structures of the vertebra (such as lamina, spinous process and posterior arch) are not fully dissected, the guiding template fails to stick closely to the posterior bony surface of the lamina; or the navigation template glides, or the holding is unstable, all theses will lead to the deviation of screw placement. The 3D guiding template is mainly made of photosensitive resin which is brittle and easy to crack, and it will be deformed and invalid when disinfected at high temperature [10].

**Fig 8:**
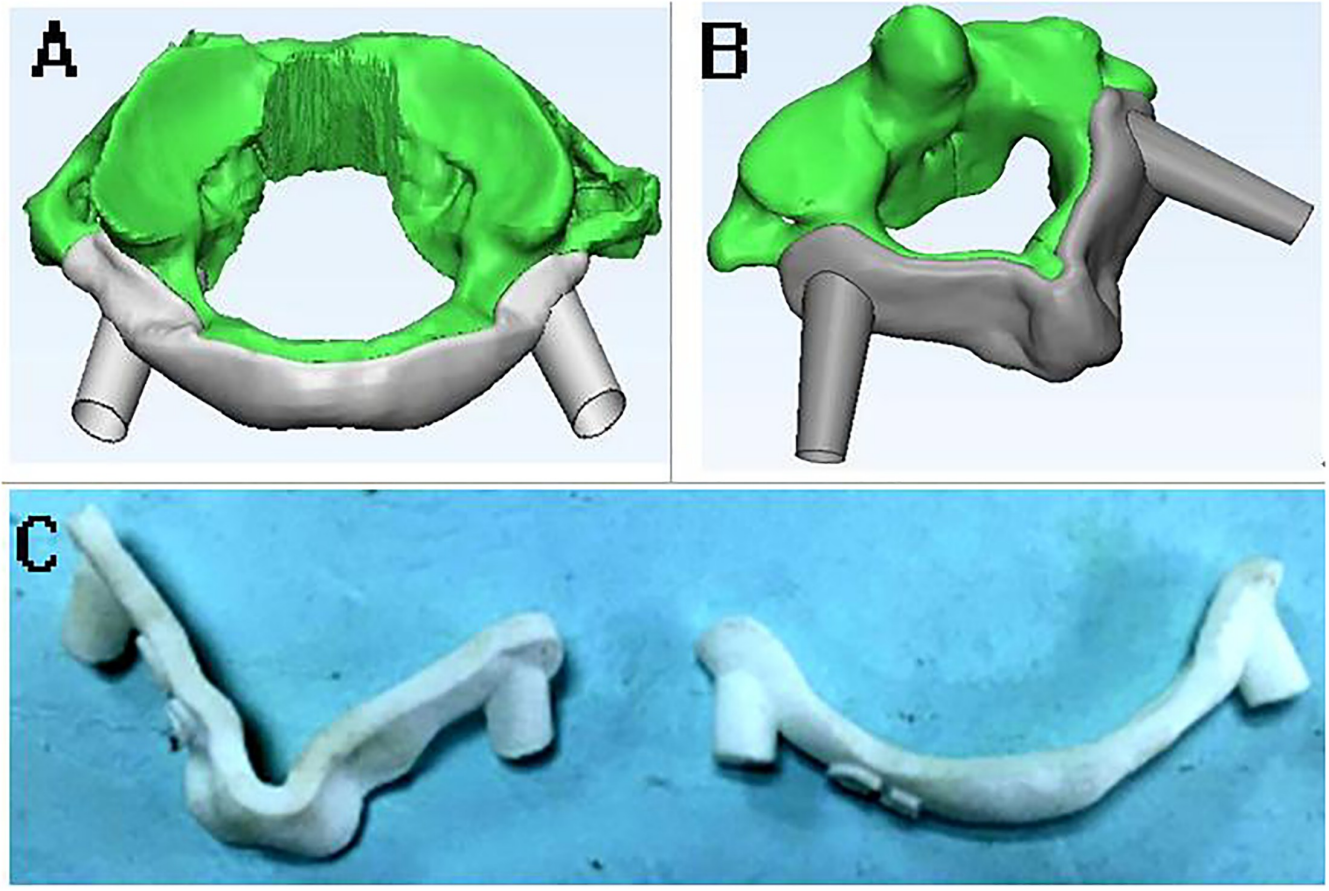
Conventional 3D printing navigation template. **(A-B)** Design of traditional guiding template. **(C)** Samples of traditional guiding template [14].

At present there are some design flaws in the conventional 3D printing navigation templates (Fig 8A–8C). First of all, the guiding channel is at the left and right ends of the template. Because of the thick neck muscles, the deep target site and narrow operating field, there are difficulties of placing the navigation template when the operation requires space on both sides. This inevitably requires a wider stripping of more soft tissue, resulting in increased soft tissue injury and bleeding. Second, the length of the guiding channel is not easy to design. If the channel is too long, the drill bit will easily have friction with the channel, resulting in vibration and displacement of the template; if the channel is too short, the drill bit will sway inside the channel, so that the tapped screw channel cannot be exactly in accordance with the planned direction. Finally, the navigation template is small in size but the surgical target site is deep, so that the guiding template is not easy to hold fixed, and it is prone to shift. This reason causes the change of the guiding channel direction which is not easy to observe, especially some slight oscillations and displacements, resulting in deviation of the screw placement. Jiang et al [11] implanted 304 cervical pedicle screws in 39 patients with 3D printing navigation templates assisted, including 287 screws at grade 0 (94.4%), 15 screws at grade I (4.9%), and 2 screws at grade II (0.7%), without any screws at grade III.

In view of the above deficiencies, we have redesigned a new type of navigation template which has not been reported in literature so far, and improved these defects. The novel navigation template canceled the original design of the screw guiding channel, but only retained the position of the screw hole; and replaced the bilateral screw guiding channels with an inward shifting navigation pole. During the operation, the screws were implanted in the screw hole on the navigation template. The navigation pole/ handrail was held to keep the navigation template stable which ensured the direction of drilling and screw implanting parallel to the navigation pole by observing and monitoring from different angles.

In the current study, we found that the novel patient-specific 3D printing navigation templates with long inward shifting guiding pole had advantages of more convenient, safer and more accurate when assisting screw placement. (a) After canceling the screw channel in the original design, and only remaining the screw hole, this design kept some flexibility with partial restriction. While indicating clearly the screw implanting point, the chances of adjusting the screw channel subjectively and manually were maintained when the channel was poor. (b) The longer navigation pole would have a larger swing amplitude when the guiding template was displaced or oscillated. By observing the change of the navigation pole, the position change of the navigation template could be more sensitively detected. In this way, it was beneficial for preventing displacement, reducing the screw implanting fault and improving the accuracy of the operation; (c) The inward shifting guiding pole was completely parallel to the screw channel of the original navigation template. The guiding pole was used as a reference for the screw implanting direction. By intraoperative multi-angle observation and monitoring, the direction of screw placement was kept to parallel with the screw pole and the correct screw channel was achieved; (d) A longer navigation pole that acted as a handrail at the same time to be held and secure the navigation template. When the direction of the drill bit was not parallel with the guiding pole, there would be a situation where the drill bit collided with the bottom of the guiding template and generate vibration. At this time, the handrail helped better maintain the stability of the navigation template and reduced its swing and displacement; (e) The inward shifting design of the double-side guiding pole could effectively reduce the volume of the navigation template, thereby minimizing the need for surgical space and soft tissue dissection, reducing bleeding and improving the safety of the operation. Therefore, the screw placement faults could be basically avoided during the operation, as long as attention is paid to exposing the posterior part of the vertebral body, dissecting the soft tissue completely, keeping the guiding template closely adhered to the bone surface, and maintaining the stability of the guiding template.

According to relative references, with the help of 3D printing guiding templates, the operation time, blood loss, screw placement time, and intraoperative fluoroscopy times were significantly less compared with the traditional manual screw placement operation. And the accuracy and safety were obviously improved. In our current study, the ideal screw channel data of the preoperative design, had no statistically differences in the transverse angle, the sagittal angle and the screw insertion point, compare with the postoperative CT scan data of the actual screw channel. In addition, postoperative CT scans also showed that up to 97.91% screw channels of all reached the Kawaguchi criterion of grade 0. It could be seen that the use of the new 3D printing navigation templates to assist the screw placement had a high accuracy; at the same time, it suggested that this method is an effective, safe and effective auxiliary implanting screw method, which would result in an actual screw channel that is highly compatible with the theoretically preset ideal screw channel. These are consistent with the results of previous reports [12,13, 14].

The following precautions should be noted when applying 3D print navigation templates for surgery: (a) Pay attention to completely remove the soft tissue attached to the posterior structure of the atlantoaxial (posterior arch, lamina and spinous process), and hold the navigation pole/handrail stably to ensure the contact surface of the guiding template is close to the bony surface; (b) Avoid the deviation of screw placement caused by the displacement of guiding template and navigation pole. Use the probe to confirm the bone integrity of the bottom and four walls of the screw channel during the operation. Take routine C-arm fluoroscopy to check the screw position; (c) High-temperature disinfection will lead to deformation and lost effectiveness of the navigation template, so epoxy ethane sterilization or low-temperature plasma disinfection is used for preoperative disinfection; (d) Since the guiding template is made of photosensitive resin which is fragile, the operation has to be gentle in surgery; (e) Before designing the screw hole of the navigation template, the drill bit size should be considered, which means the diameter of the screw hole need to be slightly larger than drill bit. At the same time, the navigation pole should be used as a reference, and the direction of the drill bit should be parallel with the pole in surgery. These steps can reduce the collision, cutting and friction between the drill bit and the base of the navigation template.

## Conclusion

The current study applied a new type of 3D printing navigation template to assist pedicle screw placement in atlantoaxial surgery, which was in accordance with the individualized characteristics of the pedicle. This method was found to have high accuracy and safety, which was beneficial to reduce the risk of operation and has a good clinical application prospects. However, as a preliminary experiment, the sample size was limited. A larger sample size of clinical application is needed in the future to further prove the accuracy and efficacy of this type of the navigation template.

## Supporting information

S1 Dataset The origin dataset of the manuscript. (XLS)
